# Assessment of Thiol/Disulfide Homeostasis and Its Association With Clinical Manifestations in Behcet’s Disease

**DOI:** 10.7759/cureus.95644

**Published:** 2025-10-29

**Authors:** Seyma Demirci, Merve Kaya, Özcan Erel, Deniz Demirseren

**Affiliations:** 1 Dermatology, Sculpture Clinic, Private Practice, Istanbul, TUR; 2 Dermatology, Health Sciences University, Ankara Bilkent City Hospital, Ankara, TUR; 3 Biochemistry, Yıldırım Beyazit University, Ankara, TUR

**Keywords:** behcet’s disease, disulfide, oxidative stress, thiol, thiol homeostasis

## Abstract

Behcet’s disease (BD) is a multisystemic inflammatory vasculitis characterized by recurrent episodes of exacerbation and remission, with unpredictable duration and system involvement in each episode. Although previous studies have shown the role of oxidative stress in the pathogenesis of BD, the relationship between the clinical features of Behcet’s disease and oxidative stress has not been fully studied to date. This study aims to assess the parameters of dynamic thiol disulfide homeostasis (DTDH) and their relationship with different clinical characteristics of disease in BD patients. Fifty patients with BD and 54 age and gender-matched healthy controls were included in the study. Disease activity was measured by the BD Current Activity Form. The mean total thiol (TT) levels were higher in BD patients compared to controls (p=0.04). The native thiol (NT) levels were lower in patients with BD compared to healthy individuals (p=0.014). Disulfide levels, disulfide/NT, and disulfide/TT levels were similar in both groups (all, p>0.05). No significant differences in DTDH parameters were observed based on the presence or absence of erythema nodosum, papulopustular lesions, venous thrombosis, arthritis/arthralgia, neurological involvement, pathergy test positivity, or HLA-B51 status (all, p>0.05). We found lower NT levels in patients with genital ulcers or eye involvement compared to those without these manifestations (p=0.036 and p=0.003, respectively). We observed similar DTDH parameters in patients with and without active disease, and in patients with and without current medical therapy (all, p>0.05). In addition, we found a negative relationship between disease duration and TT and NT levels (all, p<0.05). In conclusion, we demonstrated an imbalance of DTDH in the patients with BD, supporting the hypothesis that oxidative stress may contribute to the etiopathogenesis and progression of the disease.

## Introduction

Behcet’s disease (BD) is a chronic, relapsing, and remitting multisystemic vasculitis that typically presents with orogenital ulcers and skin lesions as well as involvement of the eye, musculoskeletal system, central nervous system, urogenital tract, vascular beds, gastrointestinal tract, and cardiopulmonary system, leading to a cause of morbidity, hospitalization, and mortality [1]. BD is mainly seen in countries along the historical Silk Road, including Turkey, Iran, Saudi Arabia, Iraq, Italy, and Israel, and also in regions with a large number of immigrants from those countries [2]. The etiopathogenesis of disease still remains uncertain, but it is suggested that endothelial destruction and environmental factors, including microbial exposure, humoral and cellular immunity abnormalities, may play a significant pathogenic role in the development and progression of the disease in genetically susceptible individuals [1]. It has been demonstrated that oxidative stress may increase in BD due to excessive production of reactive oxidant species (ROS) produced by activated neutrophils during the inflammatory response, and also decreased activity of antioxidant defense systems, including catalase, superoxide dismutase, glutathione peroxidase, which may lead to tissue damage and endothelial dysfunction in BD [3]. It is still a matter of debate whether oxidative stress is the primary inducing mechanism or a nonspecific consequence for tissue damage and endothelial dysfunction, and is also a nonspecific indicator of inflammation in BD [4]. Despite numerous studies published in the literature, the precise role of oxidative stress in the pathogenesis of BD has not been fully established.

Thiols are sulfur-containing organic compounds found in the structure of proteins such as albumin, cysteine, homocysteine, taurine, glutathione, cysteinylglycine, gamma-glutamylcysteine, and are involved in various intracellular activities, including antioxidant defense, which can prevent tissue damage by minimizing the toxic effects of oxidants [5]. Under conditions of oxidative stress, thiol groups in sulfur-containing amino acids within proteins can undergo an oxidation reaction due to increased levels of oxidants and reversibly form a covalent bond named disulfide bonds [6]. Dynamic thiol disulfide homeostasis (DTDH) at the cellular level is maintained by reducing the formed disulfide bonds to thiol groups, leading to increased thiol reserves. Studies have reported that imbalance of DTDH can be related with autoimmune conditions, malignancies, vasculitis and cardiovascular diseases [7-9]. Although previous studies have demonstrated the role of oxidative stress in the pathogenesis of BD, its relationship with the clinical manifestations of the disease has not yet been investigated [10,11]. The aim of this study is to evaluate the balance of DTDH in BD and to investigate the relationship between DTDH biomarkers and different clinical characteristics of BD.

## Materials and methods

Study design and patient selection

This study was conducted between December 2018 and December 2019 in the Department of Dermatology of Ataturk Training and Research Hospital, Ankara, Turkey. Fifty patients with BD and 54 age- and gender-matched healthy control participants were included in the study. BD was diagnosed according to the revised criteria reported by the International Study Group for Behcet’s Disease [12]. Patients scoring ≥4 points on the following scores and symptoms were diagnosed as having BD: 2 points for ocular lesions or genital ulcers or oral ulcers, and 1 point for skin lesions or neurological manifestations or vascular manifestations. Participants with acute or chronic infections, malignancy, pregnancy, or within 1 year postpartum, a history of allergic diseases, chronic kidney disease, liver disease, chronic inflammatory conditions, a history of concomitant autoimmune diseases, metabolic disorders including diabetes mellitus or thyroid disorders, cardiovascular or neurological diseases, or alcohol abuse were excluded from the study. All research procedures were evaluated and accepted by the Yıldırım Beyazıt University Clinical Research Ethics Committee and were conducted in agreement with the ethical standards specified in the Declaration of Helsinki. Written and verbal informed consent was obtained from all participants prior to their participation in this study.

Clinical and demographic characteristics, including age, gender, previous medication, duration of the disease, family history for BD and consanguinity, and the first manifestation of disease, were obtained from patients’ files. Dermatological examinations, including the presence and frequency of oral ulcers, genital ulcers, papulopustular and nodular lesions, and other skin conditions such as pyoderma gangrenosum, erythema multiforme, pernio-like skin lesions, neutrophilic eccrine hidradenitis, Kaposi’s sarcoma, extra genital ulcers, and lichen planus, were recorded and evaluated. Systemic involvements, including arthralgia and arthritis, and gastrointestinal, neurological, vascular, and ocular manifestations were also evaluated. Disease activity was measured by Behcet’s Disease Current Activity Form (BDCAF), which assesses the absence or presence of 12 clinical characteristics for the last 4 weeks, including oral ulcers, genital ulcers, headache, skin pustules, erythema, arthritis and arthralgia, gastrointestinal involvement, new ocular involvement, new neurological involvement, and new major vessel involvement [13]. The BDCAF score varies from 0 to 12 points and was evaluated according to the perspectives of both patients and physicians.

Sample collection

Blood samples were drawn from the antecubital vein after 12 hours of fasting and were centrifuged at 3000 rpm for 10 min to separate the serum. Serum samples for thiol disulfide homeostasis markers were stored at −80°C until the analyses were completed. Serum native thiol (NT), total thiol (TT), and disulfide levels were determined with the method developed by Erel and Neselioglu [14]. Briefly, reducible disulfide bonds in the serum were reduced to functional thiol groups with sodium borohydride, and residual sodium borohydride was removed using formaldehyde to prevent interference. Total thiols, consisting of both native and reduced thiols, were then quantified through reaction with Ellman’s reagent (5,5’-dithiobis (2-nitrobenzoic acid), DTNB), which produces a yellow-colored product measurable by spectrophotometry. Native thiol concentration was measured directly, and dynamic disulfide levels were calculated as half of the difference between TT and NT. Finally, disulfide/TT, disulfide/NT, and NT/TT ratios were calculated to provide a comprehensive assessment of redox status.

Statistical analysis

All analyses were performed on SPSS version 27.0 (IBM Corp., Armonk, NY, USA). To test the normality of the data distribution, Kolmogorov-Smirnov and Shapiro-Wilk normality tests were applied. Data are given as mean ± standard deviation (SD) or median (minimum-maximum) for continuous variables, and as numbers and frequency (percentage) for categorical variables. Normally distributed variables were analyzed with the independent samples t-test. Non-normally distributed variables were analyzed with the Mann-Whitney U test. Categorical variables were analyzed with the chi-square tests or Fisher’s exact tests, as appropriate. Pearson and Spearman correlation analyses were performed to assess the relationships between significantly associated parameters. A value of p<0.05 was considered statistically significant.

## Results

A total of 104 participants, 50 patients, and 54 healthy subjects were included in the study. Patients and healthy controls were similar in terms of age and gender (all, p>0.05). The mean age in the patient group was 39.00 ± 12.54 years. The mean age at time of diagnosis was 31.22 ± 8.29 years, and the mean disease duration was 7.36 ± 7.74 years in patients. In the family history, BD was revealed in 16 patients (32%) and consanguinity in 17 patients (34%). Active disease was observed in 29 (58%) patients. 27 (57.4%) patients had received medical therapy. HLA-B51 positivity was determined in 27 (54%) patients. The number of patients with clinical manifestations was as follows: 35 (70%) patients had arthritis/arthralgia, 33 (66%) patients had genital ulcers, 23 (46%) patients had erythema nodosum, 23 (46%) patients had papulopustular lesions, 14 (28%) patients had ocular involvement, 14 (28%) patients had neurological manifestations. Demographic and clinical characteristics of patients with BD are shown in Table 1.

**Table 1:** Demographic and clinical characteristics of patients with Behcet’s disease HLA-B51: Human Leukocyte Antigen-B51

Parameter	Value
Age (years)	39.00 ± 12.54
Gender (female: male) n (%)	28:22 (56%:44%)
Mean age at the diagnosis (years)	31.22 ± 8.29
Disease duration (years)	7.36 ± 7.74
The presence of family history with Behcet’s disease n (%)	16 (32%)
The presence of family history with consanguinity n (%)	17 (34%)
Active disease n (%)	29 (58%)
Current medication n (%)	27 (57.4%)
HLA-B51 positivity n (%)	27 (54%)
Presence of clinical features n (%):	
Oral ulcers	50 (100%)
Genital ulcers	33 (66%)
Erythema nodosum	23 (46%)
Papulopustular lesions	23 (46%)
Positivity of pathergy test	16 (32%)
Ocular involvement	14 (28%)
Superficial vein thrombosis	6 (12%)
Deep vein thrombosis	3 (6%)
Arthritis/arthralgia	35 (70%)
Central nervous system involvement	14 (28%)

The mean value of TT levels was 508.95 ± 52.11 µmol/L in the patient group, and 528.11 ± 40.47 µmol/L in the controls (p=0.04). The NT levels were significantly lower in patients (468.14 ± 53.01 µmol/L) compared to the controls (490.67 ± 36.33 µmol/L) (p=0.014). Although disulfide levels were slightly higher in patients, this finding was not statistically significant (p=0.247). Disulfide/NT and disulfide/TT levels were found to be similar in both groups (p=0.108, p=0.104, respectively). The comparison of thiol homeostasis parameters in both groups is shown in Table 2.

**Table 2:** Thiol and disulfide parameters of the participants Data are presented as median (minimum–maximum) for the Mann-Whitney U test and as mean ± standard deviation for the independent samples t-test. A value of p<0.05 was considered statistically significant.

Parameter	Behcet’s Disease (n=50)	Healthy Subjects (n=54)	p-value
Total Thiol (µmol/L)	508.95 ± 52.11	528.11 ± 40.47	0.04
Native Thiol (µmol/L)	468.14 ± 53.01	490.67 ± 36.33	0.014
Disulfide (µmol/L)	21.02 (4.30-80.55)	18.5 (3.05-35.25)	0.247
Disulfide/Total Thiol	0.038 (0.01-0.16)	0.035 (0.01-0.06)	0.104
Disulfide/Native Thiol	0.042 (0.01-0.24)	0.038 (0.01-0.07)	0.108

There were no significant differences in thiol metabolism parameters based on the presence of erythema nodosum, papulopustular lesions, venous thrombosis, arthritis/arthralgia, neurological involvement, HLA-B51, or pathergy test positivity (all, p>0.05). The mean value of NT in the patients with genital ulcers was lower than that of those without genital ulcers (487.63 ± 38.90 µmol/L vs. 458.10 ± 56.93 µmol/L, p=0.036). The median value of NT was found to be 427.7 (346.5-505.3) µmol/L in patients with ocular involvement and 477.3 (332.8-571.5) µmol/L in patients without ocular manifestation (p=0.015). The comparison of clinical manifestations and thiol homeostasis parameters in patients is presented in Table 3. No significant differences were observed between patients with active and inactive disease regarding thiol metabolism parameters (all, p>0.05) (Table 4). Furthermore, thiol and disulfide levels were found to be similar between patients receiving medical therapy and those not undergoing treatment (all, p>0.05) (Table 5). There were negative correlations between duration of disease and TT (r=-0.330, p=0.010) and NT levels (r=-0.345, p=0.014) (Table 6).

**Table 3:** Comparison of clinical manifestations and thiol homeostasis parameters in patients HLA-B51: Human Leukocyte Antigen-B51. Data are presented as median (minimum–maximum) for the Mann-Whitney U test and as mean ± standard deviation for the independent samples t-test. A value of p<0.05 was considered statistically significant.

Clinical Manifestations	Total Thiol	Native Thiol	Disulfide	Disulfide/Total Thiol	Disulfide/Native Thiol
Genital ulcers					
Absent (n=17)	527.03±46.97	487.63±38.90	21.20 (6-32.10)	0.03 (0.01-0.06)	0.04 (0.01-0.07)
Present (n=33)	499.63±52.84	458.10±56.93	20.85 (4.30-80.55)	0.03 (0.01-0.16)	0.04 (0.01-0.24)
p-value	0.078	0.036	0.935	0.525	0.559
Ocular involvement					
Absent (n=36)	521.05 (430.9-619.8)	477.3 (332.8-571.5)	21.38 (4.3-80.55)	0.04 (0.01-0.16)	0.04 (0.01-0.24)
Present (n=14)	455.5 (387.6-551.3)	427.7 (346.5-505.3)	16.75 (6-26.05)	0.03 (0.01-0.05)	0.04 (0.01-0.06)
p-value	0.001	0.003	0.044	0.280	0.290
HLA-B51					
Positive (n=27)	509.9±47.14	472.94±43.80	19.05(4.3-32.1)	0.04 (0.01-0.07)	0.04 (0.01-0.08)
Negative (n=23)	507.82±58.47	462.50±62.69	21.4(8.5-80.55)	0.04 (0.02-0.16)	0.04 (0.02-0.24)
p-value	0.892	0.494	0.209	0.189	0.195

**Table 4:** Comparison of thiol and disulfide parameters based on disease activity in patients Data are presented as median (minimum–maximum) for the Mann-Whitney U test and as mean ± standard deviation for the independent samples t-test. A value of p<0.05 was considered statistically significant.

Parameter	Inactive (n=21)	Active (n=29)	p-value
Total Thiol (µmol/L)	510.40 (388-620)	516.80 (421-620)	0.212
Native Thiol (µmol/L)	468 (346.5-571.5)	482.3 (332.8-571.5)	0.163
Disulfide (µmol/L)	21.40 (5.60-32.10)	20.85 (4.30-80.55)	0.701
Disulfide/Total Thiol	0.04 (0.01-0.07)	0.03 (0.01-0.16)	0.523
Disulfide/Native Thiol	0.04 ± 0.02	0.04 ± 0.03	0.737

**Table 5:** Evaluation of thiol metabolism parameters based on active medication use in patients Data are presented as median (minimum–maximum) for the Mann-Whitney U test and as mean ± standard deviation for the independent samples t-test. A value of p<0.05 was considered statistically significant.

Parameter	Patients without Treatment (n=23)	Patients Receiving Treatment (n=27)	p-value
Total Thiol (µmol/L)	514.75 (388-569)	509.73 (421-620)	0.914
Native Thiol (µmol/L)	471.30 (346.5-526.6)	468 (332.8-571.5)	0.683
Disulfide (µmol/L)	20.02 (4.30-31.75)	21.35 (6.35-80.55)	0.277
Disulfide/Total Thiol	0.03 (0.01-0.06)	0.03 (0.01-0.16)	0.591
Disulfide/Native Thiol	0.04 ± 0.01	0.05 ± 0.04	0.300

**Table 6:** Correlation between disease duration and thiol homeostasis parameters in Behcet’s disease patients A value of p<0.05 was considered statistically significant.

Parameters	r value	p-value
Total Thiol	-0.298	0.035
Native Thiol	-0.343	0.015
Disulfide	-0.094	0.515
Disulfide/Total Thiol	-0.155	0.282
Disulfide/Native Thiol	0.200	0.164

## Discussion

BD is a systemic inflammatory vasculitis affecting vessels of different sizes, types, and localization, and is characterized by recurrent periods of exacerbation and remission, with unpredictable duration for each organ and for each attack [2]. Although it can be seen at any age, the mean age of onset is mainly between 30 and 40 years, with male dominance [15]. We found the mean age at diagnosis of the disease to be 31 years and the male-to-female ratio to be 0.78:1. This gender difference may be related to the sample size and the geographical variation where our study was conducted. We also demonstrated that 32% of patients had a family history of BD and 54% of patients presented with HLA-B51 positivity. BD is commonly sporadic, but family clustering has been shown in previous studies. Family clustering has been thought of as evidence supporting genetic susceptibility in BD, especially in patients of Turkish, Israeli, and Korean descent [16]. A meta-analysis collecting data from 78 studies with 4,800 patients with BD and 16,289 healthy individuals demonstrated that the odds ratio of having HLA-B5/B51 alleles to develop the disease was 5.78 [17]. However, genetic factors have not been able to completely clarify the etiopathogenesis of BD. Histopathological studies have revealed that vascular endothelial damage was associated with neutrophil and T-lymphocyte predominant inflammatory infiltration [18]. Additionally, endothelial dysfunction mediates vascular inflammation, thrombosis, and increased oxidative stress in patients with BD [19]. Oxidative stress is also increased through chronic or active inflammatory mechanisms, including increased neutrophil functions. A decrease in the effectiveness of the antioxidant defense system and overproduction of ROS and lipid peroxides have been shown in BD pathogenesis [19-21]. Yazici et al. demonstrated increased plasma myeloperoxidase and advanced oxidation protein products in patients with BD, especially in the active state, compared to healthy individuals [20]. Köse et al. found decreased endogenous free radical scavenging enzymes, including catalase, superoxide dismutase, and glutathione peroxidase in patients with BD [21]. Becatti et al. showed in 98 patients with BD and 70 healthy controls that the structural and functional modification of fibrinogen were related to increased ROS and activated neutrophils, suggesting thrombus formation and BD-related manifestations [22]. Trace elements also function as cofactors for antioxidant enzymes. In patients with BD, erythrocyte selenium, plasma iron, manganese, and zinc levels were found to be decreased, while plasma copper, erythrocyte zinc, and manganese levels were increased [23]. Furthermore, plasma concentrations of potent non-enzymatic antioxidants, including vitamins A, C, E, and β-carotene, were found to be low in patients with BD [23]. These findings indicate that deteriorated oxidant/antioxidant equilibrium may contribute to the pathogenesis of BD as well as its morbidity and mortality.

The DTDH has a critical and vital role in apoptosis, detoxification, cellular signaling mechanisms, signal transduction, regulation of enzyme activities, immunity, inflammation, as well as antioxidant protection [7]. Thiols are essential physiological antioxidant buffers and are responsible for 52.9% of the total antioxidant capacity in healthy individuals [24]. The thiol groups of sulfur-containing amino acids in proteins are the main targets of ROS. Thiol oxidation and disulfide bond formation, a dynamic and reversible process, act as an early cellular response to ROS exposure. These disulfide bond-containing structures can be reduced back to thiol groups through the antioxidant systems in the organism to maintain a dynamic thiol-disulfide balance. Measurement of DTDH metabolites is critical to evaluate the intracellular oxidation state and conditions associated with oxidative stress, including hypertension, myocardial infarction, cancers, autoimmune diseases, as well as different physiological, pathological, and biochemical processes [7-9]. Thiol groups (-SH) serve as critical antioxidants and are widely utilized as biomarkers of oxidative stress. Quantification methods include Ellman’s reagent colorimetric assays, fluorescence-based assays, high-performance liquid chromatography, mass spectrometry, and electrochemical techniques. The accuracy of these methods can be significantly influenced by endogenous reducing components such as hemoglobin, lipids, and bilirubin, which may interfere with thiol detection and lead to erroneous estimations. In our study, however, hemoglobin, lipemic, and uremic plasma samples, as well as ethylenediaminetetraacetic acid (EDTA), citrate, and oxalate, do not interfere with the assay, whereas bilirubin is known to interfere with the assay [14]. While reductive thiol levels have been measured since 1979, reducible disulfide can now be measured using a reliable, inexpensive, fully automated method recently developed by Erel and Neselioglu [14].

Few studies have shown the change of DTDH biomarkers in patients with BD. Cetin et al. demonstrated decreased NT, TT, and disulfide levels in 42 patients with BD compared to controls. They also showed NT and TT levels were lower in patients with active disease compared to inactive patients [25]. Sandikci et al. showed decreased NT, TT levels, and NT/TT ratio and increased disulfide/NT and disulfide/TT values in 150 BD patients compared to those of 100 healthy individuals NT [11]. We found that the TT levels, indicating the total antioxidant status, were lower in the patient group compared to the controls, and NT levels, indicating the antioxidant status, were also lower in the patient group. We also found slightly increased disulfide levels, not statistically significant, in our patient group. These findings suggest that oxidative stress damage and alteration in thiol disulfide homeostasis, and increased oxidative damage and inadequacy of antioxidant mechanisms may occur in the pathogenesis of BD. Imbalance in the TDH may contribute to endothelial dysfunction and tissue damage in the development and progression of the disease. We showed similar DTDH levels in patients with and without active disease, or in patients who received and did not receive medication. Consistent with our study, Sandikci et al. demonstrated similar thiol and disulfide levels in active and inactive BD patients [11]. Our results indicate that thiol disulfide balance is not affected by disease activity and current medical therapy. These results may be due to the absence of a reliable diagnostic indicator showing the activity of the disease, and also, our results suggest that the TDH markers cannot be used to determine disease activity. This also may be due to the heterogeneous clinical features of our study group, as the disease progresses with relapse and remissions. In addition, this was the first study to examine the relationship between DTDH markers and clinical findings, including cutaneous involvement, neurological involvement, joint involvement, HLA-B51 status, and pathergy test result. We found that patients with or without these manifestations had similar results for TDH biomarkers. We showed decreased NT levels in patients with genital ulcers or patients with ocular involvement compared to those without these manifestations. Balbaba et al. showed decreased NT, TT levels, and NT/TT ratio and increased disulfide levels, disulfide/NT, and disulfide/TT ratio in 20 ocular-active BD patients compared to 20 ocular-inactive patients [10]. In contrast, Sandikci et al. showed no significant differences between the uveitis-positive and uveitis-negative patients or between the vascular involvement-positive and negative patients in DTDH biomarkers [11]. While an increase in antioxidant activity may occur in the early stages of the oxidation process, as a result of prolonged oxidative changes, reduced antioxidant capacity in tissues and body fluids of patients may occur in the late stages of the disease. The decrease in NT levels in patients with genital ulcer or ocular involvement may be attributed to the impairment and insufficiency of antioxidant mechanisms in these individuals. Furthermore, we observed a significant negative correlation between the duration of the disease and TT and NT values. These results show that antioxidant defense mechanisms were insufficient due to the prolonged exposure to intense oxidative stress.

The interplay between systemic oxidative stress and inflammation is a fundamental aspect of many chronic diseases, including BD. Oxidative stress arises when there is an imbalance between the production of ROS and the body's antioxidant defenses, leading to cellular damage. This damage can activate inflammatory pathways, resulting in the release of pro-inflammatory cytokines such as interleukin-6 (IL-6) and tumor necrosis factor-alpha (TNF-α). These cytokines, in turn, can stimulate the production of more ROS, creating a cycle that exacerbates tissue injury and disease progression. In BD, this cycle may contribute to endothelial dysfunction and tissue damage, highlighting the importance of understanding the molecular mechanisms underlying this interplay for developing targeted therapeutic strategies [26].

This study has several limitations. The primary limitation of this study is its cross-sectional design, which prevents us from determining whether these findings are the cause or consequence of BD. Secondly, TDH biomarkers can be affected by conditions such as nutritional status, body mass index, sleep routine, lifestyle habits, and smoking [27]. We could not exclude these factors in our study. Finally, our study also had a relatively small sample size.

## Conclusions

In conclusion, we found an imbalance of thiol disulfide homeostasis in the patients with BD, supporting the idea that oxidative stress may contribute to the etiopathogenesis and progression of the disease. This was the first study to report the association with DTDH parameters and various clinical features of BD. The determination of DTDH may provide insight into intracellular oxidative status and serve as a key therapeutic target for oxidative stress in the management of BD.
